# Psilocybin microdosing does not affect emotion-related symptoms and processing: A preregistered field and lab-based study

**DOI:** 10.1177/02698811211050556

**Published:** 2021-12-17

**Authors:** Josephine Marschall, George Fejer, Pascal Lempe, Luisa Prochazkova, Martin Kuchar, Katerina Hajkova, Michiel van Elk

**Affiliations:** 1 Department of Psychology, University of Amsterdam, Amsterdam, The Netherlands; 2 Department of Psychology, Leiden University, Leiden, The Netherlands; 3 Forensic Laboratory of Biologically Active Substances, Department of Chemistry of Natural Compounds, University of Chemistry and Technology Prague, Prague, Czech Republic; 4 Department of Experimental Neurobiology, National Institute of Mental Health, Klecany, Czech Republic

**Keywords:** Psilocybin, microdosing, psychedelics, emotion processing, interoceptive awareness, anxiety, depression, symptoms

## Abstract

**Background:**

Microdoses of psychedelics (i.e. a sub-hallucinogenic dose taken every third day) can reduce symptoms of depression, anxiety and stress according to anecdotal reports and observational studies. Research with medium to high doses of psilocybin points towards potential underlying mechanisms, including the modulation of emotion and interoceptive processing.

**Aims:**

In this preregistered study, we investigated whether psilocybin microdoses alter self-reported interoceptive awareness and whether repeated microdosing over 3 weeks modulates emotion processing and reduces symptoms of anxiety and depression.

**Methods:**

We used a double-blind, placebo-controlled, within-subject crossover design. Participants completed the Multidimensional Assessment of Interoceptive Awareness Questionnaire 1½ h after self-administering their second dose (or placebo), and the emotional go/no-go task and the shortened Depression Anxiety Stress Scale 1½ h after self-administering their seventh dose.

**Results:**

Our confirmatory analyses revealed that psilocybin microdosing did not affect emotion processing or symptoms of anxiety and depression compared with placebo. Our exploratory analyses revealed that psilocybin microdosing did not affect self-reported interoceptive awareness, that symptoms of depression and stress were significantly reduced in the first block compared with baseline, that participants broke blind in the second block and that there was no effect of expectations. Further research in a substance-naïve population with clinical range anxiety and depressive symptoms is needed to substantiate the potential beneficial effects of microdosing.

## Introduction

Microdosing has become increasingly popular over the last decade. A microdosing regimen typically entails the ingestion of a psychedelic substance at a sub-hallucinogenic dose, usually 5–10% of a standard dose. Psilocybin and lysergic acid diethylamide (LSD) are the most common psychedelics used for microdosing and users commonly follow the Fadiman protocol, which suggests that one should dose every third day to achieve optimal effects (Fadiman, 2011; Hutten et al., 2019). Anecdotal reports and observational studies suggest that microdosing can have antidepressant and anxiolytic effects (Anderson et al., 2019; Cameron et al., 2020; Fadiman and Korb, 2019; Johnstad, 2018; Kaertner et al., 2021; Lea et al., 2020; Petranker et al., 2020; Polito and Stevenson, 2019; Webb et al., 2019). Users with mental health conditions, such as anxiety and obsessive-compulsive disorder (OCD), report microdosing as a form of self-medication (Hutten et al., 2019; Johnstad, 2018).

However, the available evidence regarding the efficacy of microdosing for mental health remains inconsistent. Three of the four existing experimental studies on humans found no evidence for the alleged antidepressant and anxiolytic effects (Bershad et al., 2019; Family et al., 2020; Szigeti et al., 2021). The fourth, Hutten et al. (2020), found that 20 μg LSD increased positive mood but also anxiety. These inconsistencies may well be related to differences in study designs. The observational studies investigated the effects of microdosing longitudinally, either by gathering cross-sectional data from a subpopulation of individuals who regularly practice microdosing, or via prospective observational design that gathered data from before, during, and after a predetermined microdosing period (Bornemann, 2020). These studies did not control for psychedelic substances or dosages. In contrast, three of the four experimental studies focused only on the effects of LSD microdosing compared with placebo and investigated the acute effect of varying doses (Bershad et al.: 6.5, 13 and 26 μg; Family et al.: 5, 10 and 20 μg; Hutten et al.: 5, 10 and 20 μg). Participants of the fourth self-blinded experimental study self-administered psychedelic microdoses of their choice (i.e. primarily LSD and psilocybin), over 4 weeks. The authors assessed both acute and post-acute effects and controlled for varying dose quantities (Szigeti et al., 2021). The two experimental studies investigating the effect of microdosing on rats also reveal mixed results. Horsley et al. (2018) found a modest anxiogenic effect in the elevated-plus maze when the rats were tested 48 h after the third microdose of both psilocin and ketamine. In contrast, Cameron et al. (2019) found no effects of a 2-month dimethyltryptamine (DMT) microdosing protocol on anxiety but did find reduced immobility in the forced swim paradigm, which is considered an antidepressant-like effect, and less freezing behaviour following fear extinction training, which may reflect enhanced fear extinction.

This study aims to further reconcile some of these inconsistent findings through a combined field and lab study design. We investigated the effect of psilocybin microdosing, compared with placebo, over the course of 3 weeks, at one consistent dosage, on humans. Next to measuring self-reported changes in mood and anxiety, we explored potential underlying mechanisms of the alleged anxiolytic and antidepressant effects: emotion processing and interoceptive awareness. Previous research has shown that standard doses of psilocybin interfere with the processing of negative facial expressions and induce a bias towards positive emotions in an emotional go/no-go paradigm (Bernasconi et al., 2013; Kometer et al., 2012; Kraehenmann et al., 2015; Schmidt et al., 2012). These modulations were accompanied by enhanced positive affect. In addition, psilocybin has been shown to disrupt pre-attentive sensory-motor gating, which could allow for an influx of exteroceptive and interoceptive information (Vollenweider, 2001; Vollenweider et al., 2007). Such influx may lead to increased interoceptive awareness, which has been associated with awareness and regulation of emotional states (Füstös et al., 2012). A neurocognitive mechanism which may underlie the effects of psilocybin on emotion processing and interoceptive awareness can be found in the predictive processing framework (Clark, 2013). Here, psilocybin-induced hyper-activated 5-HT2a receptors in layer V pyramidal neurons decompose the categorical top-down predictions we have about exteroceptive and interoceptive stimuli into more diverse and fine-grained predictions (Pink-Hashkes et al., 2017). This process may in turn disrupt top-down emotion and multisensory processing.

In this study, we assessed the effects of psilocybin microdosing on mood and anxiety symptoms using the shortened Depression Anxiety Stress Scale (DASS-21; Lovibond and Lovibond, 1995), on emotion processing using the emotional go/no-go task (Erickson et al., 2005; Schulz et al., 2007) and on interoceptive awareness using the Multidimensional Assessment of Interoceptive Awareness Scale (MAIA; Mehling et al., 2012). Our decision to include the DASS-21 was influenced by the observational study by Polito and Stevenson (2019; *n* = 63), which included this measure to assess the longitudinal effect of microdosing and found significant effects for Depression and Stress subscales after 6 weeks. The authors did not report effect sizes. Our inclusion of the emotional go/no-go task was based on the double-blind, placebo-controlled study of Kometer et al. (2012; *n* = 17), who used this task to assess alterations in emotion processing under standard doses of psilocybin and found that psilocybin increased reaction time (RT) as a function of valence ( $\eta_{\text{p}}^{2}$ = 0.296). Specifically, psilocybin increased RTs more for negative and neutral go stimuli compared with positive go stimuli, thereby inducing a bias to positive stimuli. We formulated preregistered hypotheses and confirmatory analysis plans for these measures. The MAIA and other post hoc analyses are considered exploratory.

### Hypotheses and preregistration

This study was part of a larger collaborative project with researchers of different research interests and thus also included other cognitive and behavioural measures investigating the effect of microdosing on awe and art perception, temporal recalibration, creativity and bistable perception. The preregistration of the project and study-specific data can be found on the Open Science Framework (https://osf.io/cn8z4/). The study on awe and art perception has been published (van Elk et al., 2021) and the study on creativity is currently under review (see preprint: https://psyarxiv.com/emcxw/). All studies in this project used the same psilocybin-containing truffle analysis of potency and the same pool of participants, but inclusion of participants and measurement sessions differed depending on study-specific designs and methods.

We preregistered the hypotheses and analysis plans regarding the DASS-21 and emotional go/no-go task within the project preregistration. We did not preregister our hypothesis and analysis plan for the MAIA, but we wrote that we expected an increase in interoceptive awareness during the acute effect of the psilocybin microdose compared with placebo. We specified the following hypotheses in advance of the conducting the study:

H1: The acute and additive action of seven psilocybin microdoses (compared with placebo) will significantly reduce acute scores of depression and anxiety as measured by the DASS-21.
H2: The acute and additive action of seven psilocybin microdoses (compared with placebo) will significantly increase acute RT more for angry, fearful and sad facial expressions than for happy facial expressions as measured by the emotional go/no-go task.
H3: The acute and additive action of seven psilocybin microdoses on scores of the DASS-21 is mediated by increased RTs for angry, fearful and sad facial expressions.

We also preregistered that we would include block-order as a between-subjects factor in the analyses related to the above expectations. The block-order variable differentiates between those who received psilocybin in the first block, labelled the ‘Psilocybin-first’ group, and those who received placebo in the first block, labelled the ‘Placebo-first’ group. This was done to test whether effects are related to practice and are stronger in the first compared with second block.

## Methods

### Participants

Of all contacted participants (for recruitment strategy, see ‘Procedure’ section), 75 passed the screening questionnaire and signed up for the subsequent lab sessions. The screening excluded participants who are, or ever have been, diagnosed with schizophrenia, psychosis, mania or borderline or have genetically related relatives with these conditions. Moreover, we excluded individuals with substance abuse disorders and/or currently taking a medication, those with other serious physical and/or mental health issues and those who were not proficient in English. Participants interested in joining the study were asked to abstain from psychoactive substances within the 2 weeks preceding the microdosing workshop. We also asked participants to comply with specific behavioural guidelines during their 2-month participation in our study. Specifically, we asked that participants abstain from psychoactive substances, other than their microdose, from the 2 weeks preceding the microdosing workshop until the end of their 2-month participation; that they self-administer their doses no more than 3 h and no less than 30 min before their scheduled lab session, and self-administer at least five doses per block and that they remain blind to their condition.

Of 75 initial participants, 63 completed the baseline measures, 68 completed session 1 (S1), 61 completed session 2 (S2), 59 completed session 3 (S3) and 56 completed session 4 (S4). We did not collect information as to their motivations for dropping out of the study. Thus, 58 participants completed measures at both S1 and S3, 55 completed measures at both S2 and S4 and 49 completed measures at baseline, S2 and S4. We further excluded participants from the analysis who did not comply with the behavioural guidelines: 6 participants in S1 and S3, and 11 participants in sessions S2 and S4. These participants either consumed other psychoactive substances during the study or deviated in their microdosing schedule from acceptable conditions of dosing. The acceptable conditions include that participants should dose between 5 and 7 doses per 3 weeks, that they leave at least 1 day between doses, that they take the full doses and that they ingest their dose within 45 min to 2.5 h before each lab session. Thus, for the repeated-measures analyses further discussed below, 52 participants were included for S1 and S3, consisting of 29 females and a mean age of 29.75 (ranging from 29–60) years and 44 were included for S2 and S4, consisting of 21 females and a mean age of 30.6 (ranging from 20–60) years.

### Procedure

We recruited and screened participants who planned to attend one of three consecutive microdosing workshops co-organized by the Psychedelic Society of the Netherlands (PSN) and Microdosing.nl. At the workshops, the participants saw two presentations, the first provided by the hosts discussing anecdotal reports regarding best practices of microdosing and the second provided by the researchers outlining the project and behavioural guidelines (see ‘Workshop Materials’ at OSF https://osf.io/cn8z4/). No explicit safety instructions were provided to our participants. Participants then read the information letter and provided their written consent, completed the DASS-21 (all scales and instruments are described in more detail below) and created their own batch of seven microdoses (for information about dosage, see below). A member of the PSN then randomized the participants’ bags containing psilocybin microdoses with bags containing non-active placebo doses. At the end of the workshop, participants received two bags, each containing seven doses of either psilocybin or placebo. They were instructed to consume one bag of doses over the subsequent 3-week period, then to take a 2-week break and, finally, to consume the second bag of doses over another 3-week period. We will refer to the first 3-week period as block 1 and the second 3-week period as block 2.

**Figure 1.**
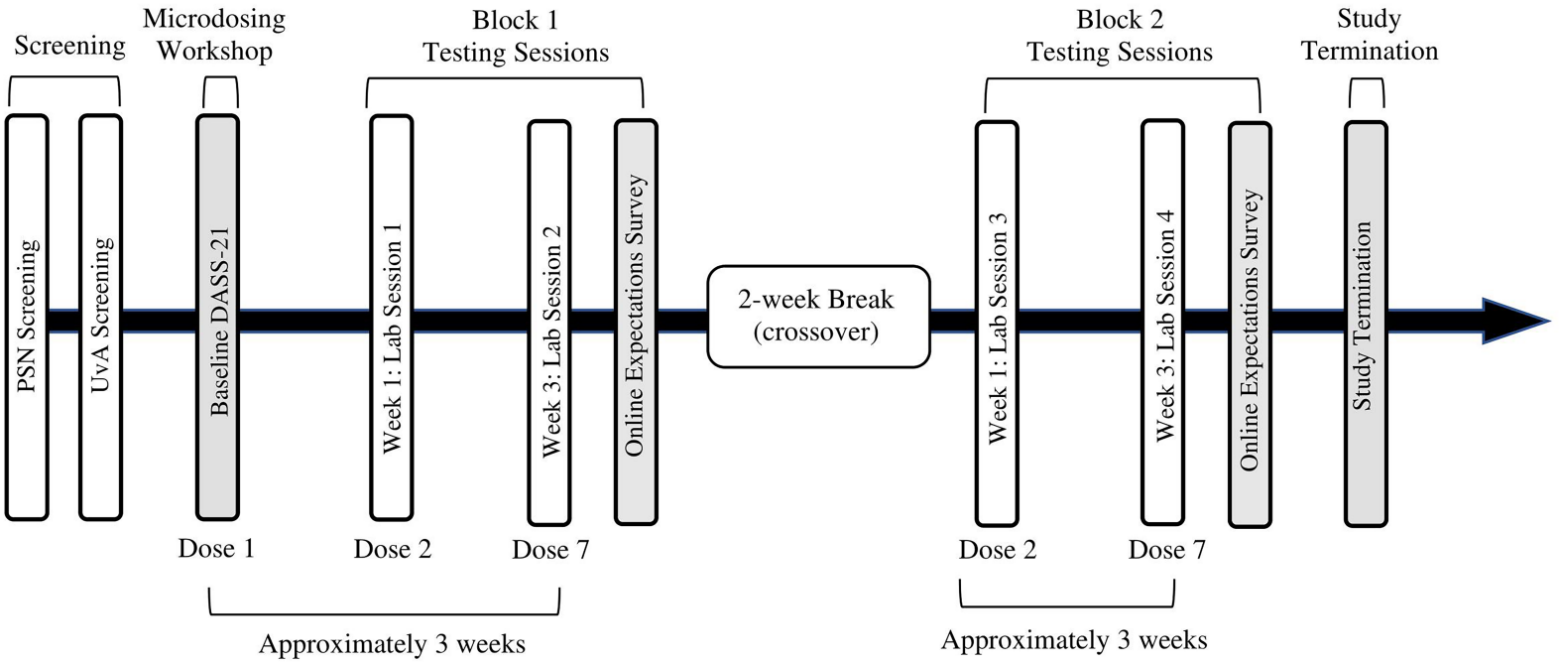
Project timeline. PSN: Psychedelic Society of the Netherlands; UvA: University of Amsterdam; MAIA: Multidimensional Assessment of Interoceptive Awareness Scale. Half of the participants took their psilocybin doses during block 1, while the other half took their psilocybin doses during block 2. The group that was not taking psilocybin doses at a given period was instead taking placebo doses. The MAIA was implemented during S1 and S3, while the DASS-21 and emotional go/no-go instruments were implemented during S2 and S4. Online questionnaires regarding condition expectations were administered in the days following the seventh dose.

The participants signed up for four lab sessions, two sessions per block. Each session took place 1.5 h after self-administering a dose, based on the finding that plasma psilocin concentration of low doses peaked around 1.5 h after ingestion (Madsen et al., 2019; Passie et al., 2002). S1 and S3 were scheduled after self-administration of the second dose per bag while S2 and S4 were scheduled after self-administration of the seventh dose per bag. The measures in this study were part of a larger test battery lasting 1 h per session. Participants completed the MAIA in S1 and S3, within the first 15 min of the session. Participants completed the DASS-21 and emotional go/no-go task in S2 and S4 within the first 20 min of the session. Table 1 and Figure 1 present an overview of the experimental sessions and the different events during the study. The test battery also included a screening at the beginning of each session to assess perceived strength of the dose effects, use of other psychoactive substances and level of tiredness on the day of testing (for complete version of the questionnaire, see https://osf.io/cn8z4/). The day following the lab sessions, participants answered an online survey about their experience of their condition to clarify whether they broke blind. The study protocol was approved by the local ethics committee at the University of Amsterdam (#2019-SP-10060). The experiment was conducted in accordance with the guidelines of the Declaration of Helsinki.

**Table 1.** Overview of timeline components.

Session	Block	Dose	Instruments
Baseline	0	0	DASS-21
S1	B1	2	MAIA
S2	B1	7	DASS-21; Emotional Go/No-Go
S3	B2	2	MAIA
S4	B2	7	DASS-21; Emotional Go/No-Go

DASS-21: Depression Anxiety Stress Scale-21; MAIA: Multidimensional Assessment of Interoceptive Awareness Scale.

### Doses

The psilocybin microdoses were created by the participants during the microdosing workshop. The doses contained 0.7 g of dried psilocybin-containing Galindoi truffles, which corresponds to around 1/10th of a medium-high dose. Participants were instructed to keep the doses in the fridge. The placebo doses contained dried non-psychoactive mushrooms and seeds to match the weight and sound of the psilocybin doses. The doses were masked using non-transparent capsules. The participants placed their seven psilocybin capsules into a plastic bag with their participant number. A member of PSN randomly labelled half of the bags with the number ‘1’ and the other half with number ‘2’, corresponding to the 3-week block at which the doses in this bag should be consumed. This member then matched bags containing the seven placebo capsules to each participant, labelling the bag with number ‘1’ if the psilocybin bag was labelled 2, and vice versa. Thus, at the end of the workshop, each participant received back one bag labelled ‘1’ and another bag labelled ‘2’ and given the instruction that bag 1 should be consumed in the first block while bag 2 should be consumed in the second block (after the 2-week break). The dose-order per participant was recorded by this PSN member and revealed to the researchers and participants only after data collection and analysis.

### Instruments and scales

#### DASS-21

The DASS-21 is a shortened version of the DASS (Lovibond and Lovibond, 1995) consisting of 21 items pertaining to the severity/frequency of depression, anxiety and stress (seven items per emotion) over the past week. The items, such as ‘I tended to over-react to situations’, are rated on a 4-point scale from 0 (did not apply to me at all) to 3 (applied to me most of the time). The scale consists of 3 subscales, namely, depression, anxiety and stress. The sum for each subscale is calculated and then doubled to allow comparison with the original 42-item scale. For this study, we chose to focus on subscales Depression and Anxiety for confirmatory analyses but included the Stress subscale in our exploratory analyses. The DASS-21 is suitable for repeated measures and has been used in both clinical and non-clinical samples. It is considered a reliable and valid measure of each subscale construct, with strong convergent validity with the original DASS, the Hospital Anxiety and Depression Scale and the Beck Depression Inventory-II (Bener et al., 2016; Henry and Crawford, 2005; Norton, 2007; Osman et al., 2012). The participants in this study completed this questionnaire at baseline during the workshop, on their seventh microdose and on the seventh placebo (S2 & S4).

#### Emotional go/no-go task

In this task, participants are instructed to respond to a ‘Go’ stimulus as fast as possible and not to respond to the ‘No-Go’ stimulus. The go and no-go stimuli are defined for the participant in the instructions at the beginning of each trial block. In this study, we used pictures of emotional faces as our go and no-go stimuli, instead of words as used by Kometer et al. (2012), because many of our participants were not native English speakers. We obtained our stimuli from the Amsterdam Dynamic Facial Expression Set of validated emotional face stimuli, which included pictures of sad, fearful, angry, happy and neutral faces (Schalk et al., 2011).

The task consisted of eight trial blocks of go/no-go emotional face category pairs: sad/neutral, neutral/sad, fearful/neutral, neutral/fearful, angry/neutral, neutral/angry, happy/neutral and neutral/happy. Here, fearful/neutral, for example, represents a block in which the go stimulus consists of fearful faces, to which participants are instructed to respond, while the no-go stimulus consists of neutral faces, to which participants are instructed to inhibit their response. One stimulus pair was presented per block and the blocks were randomized across participants. The stimuli were displayed on a computer screen of a 21-inch CRT (cathode-ray tube) monitor and participants responded by pressing the keyboard space bar. At the beginning of the task, participants completed a practice block of the neutral/fear stimulus pair, consisting of 15 go trials and five no-go trials. Participants then went on to complete eight testing blocks, in which each go stimulus category was alternated with the no-go stimulus category at a proportion of 67% and 333%, respectively. Specifically, each block consisted of 30 trials, of which 20 were go stimuli and 10 were no-go stimuli. Participants received automatic breaks between blocks and could decide for themselves when they were ready to start the next block by pressing the space bar.

**Figure 2.**
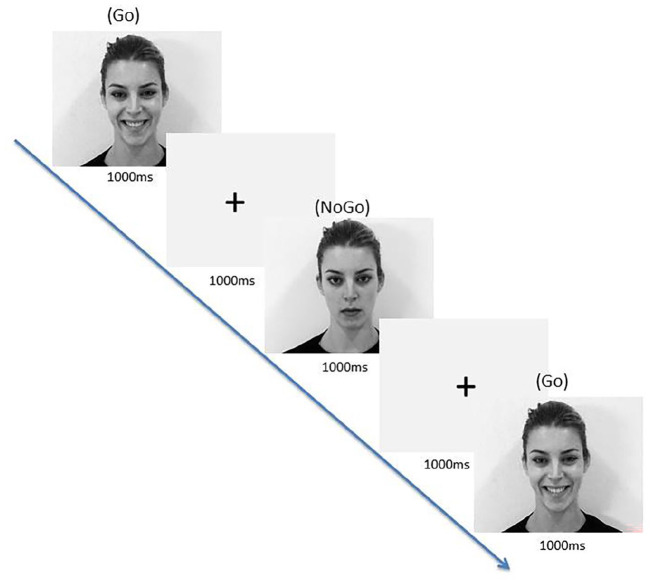
Emotional go/no-go task trial for happy ‘go’ cue with neutral ‘no-go’ cue, with response intervals of 1000 ms and interstimulus intervals of 1000 ms. The words ‘Go’ and ‘No-Go’ were not displayed to the participants; they are included in this figure to clarify the correct responses in this example.

Participants were required to respond within 1000 ms while the picture was presented on the screen. The pictures within each trial were separated by a fixation cross of 1000 ms (see Figure 2; Tottenham et al., 2011). Thus, the interstimulus interval was 1000 ms. Participants’ RTs to the stimuli were automatically registered with respect to stimulus-onset when participants pressed the space bar during a block. Participants completed this measure 1½ h after taking their seventh microdose, and their seventh placebo dose, to allow for comparison with the DASS-21 results (S2 & S4).

#### MAIA

The MAIA (Mehling et al., 2012) is a 32-item self-report questionnaire used to assess eight constructs of interoceptive body awareness. The items are rated based on how often they apply generally in daily life from 0 (never) to 5 (always). Sample items include ‘I can use my breath to reduce tension’ and ‘I notice where in my body am I comfortable’. For this study, we chose to focus on four of the eight subscales: noticing, emotional awareness, self-regulation and body listening. To assess the acute microdose effect on these subscales, participants were asked to rate the items based on to what extent the items applied to their current state of being. The MAIA is a reliable measure with appropriate convergent and divergent validity based on constructs of mindfulness and bodily awareness (Mehling et al., 2012). Moreover, it is suitable for a repeated-measures design (Bornemann et al., 2015). Participants completed this questionnaire 1½ h after taking their second microdose and their second placebo dose (S1 & S3).

#### Additional questions and questionnaires

The above instruments were administered at the start of a larger test battery lasting 1 h per session. Participants answered screening questions at each lab session to assess their adherence to the behavioural guidelines. Participants were also asked to guess their condition after each dosing block, specifically ‘In the past few weeks, do you think you were taking an active microdose?’ to which they could respond with ‘yes’, ‘no’ or ‘maybe’.

### Data processing and analyses

#### DASS-21

We summed the scores of each subscale (anxiety, depression, stress) and multiplied these sums by two, making the values comparable with the original DASS scale of 42 items. We split the data per condition (psilocybin/placebo, coded as 0/1), per session (baseline/first block/second block) and per subscale (depression/anxiety/stress). The separate subscales were analysed using both a Bayesian and frequentist repeated-measures analysis of variance (ANOVA) (rmANOVA) with condition (psilocybin or placebo) as the within-subject factors. For this and all other analyses, we used the statistical programme JASP. We expected more evidence for the alternative hypothesis, reflected in a BF_10_ > 7 and a significant main effect of condition below the *p* = 0.05 threshold of probability. The frequentist analysis was anticipated in the preregistration. We included the Bayesian analysis as well, to allow quantifying the relative evidence in favour of the null compared with the alternative hypothesis.

#### Emotional go/no-go task

For each emotion block and per participant in S2 and S4, we averaged the RTs of the correct responses and removed trials that exceeded two standard deviations (SD) from the subject’s average. This resulted in 40 participants. We omitted the blocks ‘neutral-fearful’ and ‘fearful-neutral’ due to an error in the task instructions. We then reorganized the data per group (psilocybin/placebo, coded as 0/1). The RTs were analysed using both a Bayesian and frequentist rmANOVA with condition and emotion block as within-subject factors. Here, we expected more evidence for the alternative hypothesis, reflected in a BF_10_ > 7 and a Condition × Emotion interaction effect below the *p* = 0.05 threshold of probability. This would indicate that psilocybin affects RT as a function of emotion. Pending a significant interaction effect, we planned to conduct post hoc independent *t* tests to determine which emotion is driving the interaction effect.

#### MAIA

We summed the scores per subscale. The subscales were analysed separately using both a Bayesian and frequentist rmANOVA with condition (psilocybin, placebo) as the within-subject factors. Here, we expected more evidence for the alternative hypothesis, reflected in a BF_10_ > 7 and a significant main effect of condition below the *p* = 0.05 threshold of probability.

#### Condition identification

To assess whether participants could accurately identify their condition, we used a chi^2^ analysis of the contingency table of guessed condition with accurate condition per block. Of those participants who complied with the behavioural guidelines, 46 provided a guess in block 1 and 30 provided a guess in block 2.

## Results

### Doses

A sample of the dried psilocybin-containing truffles was analysed to determine the potency. This analysis revealed an alkaloid concentration of approximately 2129.2 µg/g, which translates to approximately 1.5 mg per 0.7 g dried truffle dose administered by our participants. The details and results of this analysis can be found in the Supplemental Material (see https://osf.io/cn8z4/) and were also reported by van Elk et al. (2021) and Prochazkova et al. (2021) as these studies were all part of the larger collaborative project including the same participant sample.

### Descriptive statistics

#### DASS-21

The Cronbach’s alpha for subscale anxiety was 0.69 in block 1 and 0.49 in block 2. The Cronbach’s alpha for subscale depression was 0.79 in block 1 and 0.79 in block 2. The mean baseline scores of the Anxiety and Depression subscales were in the ‘normal’ range of symptoms severity, while the Stress scores were in the ‘moderate’ range. The mean scores for placebo and psilocybin conditions were within the ‘normal’ range of symptom severity for Anxiety and Depression subscales and in the ‘mild’ range for the Stress subscale (see Table 2; Lovibond and Lovibond, 1995).

**Table 2.** DASS-21 average subscale scores per condition.

Symptom	Condition	Mean	SD	*n*
Depression	Baseline	9.35	7.53	40
	Psilocybin	5.41	4.26	44
	Placebo	5.36	4.84	44
Anxiety	Baseline	8.05	6.27	40
	Psilocybin	3.82	3.25	44
	Placebo	5.05	4.60	44
Stress	Baseline	16.50	7.75	40
	Psilocybin	9.96	5.41	44
	Placebo	11.05	7.19	44

DASS-21: Depression Anxiety Stress Scale-21; SD: standard deviation.

The scale range for depression is as follows: 0–9 = ‘normal’, 10–13 = ‘mild’, 14–20 = ‘moderate’, 21–27 = ‘severe’, 28 + = ‘extremely severe’.

The scale range for anxiety is as follows: 0–7 = ‘normal’, 8–9 = ‘mild’, 10–14 = ‘moderate’, 15–19 = ‘severe’, 20 + = ‘extremely severe’.

#### Emotional go/no-go

**Figure 3.**
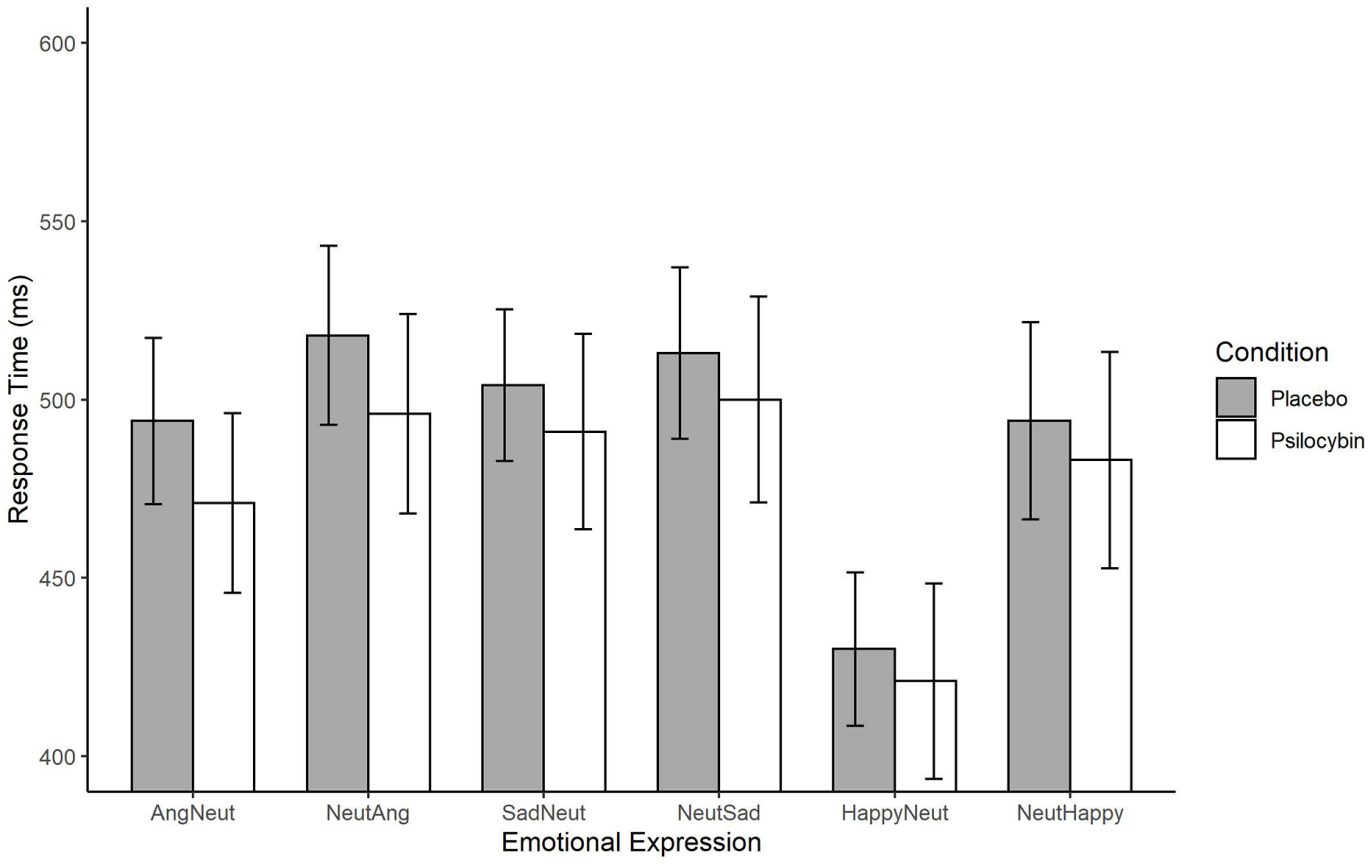
Emotional go/no-go reaction times. Ang: angry; Neut: neutral; RT: reaction time. Average reaction times per condition, with 95% confidence intervals, for each emotional ‘go’ expression. ‘AngNeut’ indicates RT to angry face stimuli embedded in neutral face trials while NeutAng indicate RT to neutral face stimuli embedded in angry face trials.

We calculated the mean RT per emotion block in each condition (see Figure 3). We also calculated response accuracy per emotion block in each condition (see Table 3).

**Table 3.** Emotional go/no-go response accuracy.

Condition	Proportion of correct responses per emotion block			
		Sad	Happy	Angry
Psilocybin	Neutral go	0.94	0.98	0.96
	Neutral no-go	0.94	0.94	0.93
Placebo	Neutral go	0.94	0.98	0.96
	Neutral no-go	0.92	0.95	0.93

Correct responses = Hits + Correct Rejections. A hit is the response to the go stimulus. A correct rejection is the lack of response to the no-go stimulus.

#### MAIA

Cronbach’s alpha per subscale per block is reported in Table 4. We calculated the averages per condition per subscale of the MAIA, which are displayed in Table 5.

**Table 4.** Cronbach’s alpha for MAIA subscales per block.

Subscale	Block 1	Block 2
Noticing	0.73	0.85
Emotional awareness	0.89	0.84
Self-regulation	0.87	0.84
Body listening	0.89	0.85

MAIA: Multidimensional Assessment of Interoceptive Awareness Scale.

**Table 5.** MAIA average subscale scores per condition.

Subscale	Condition	Mean	SD	*n*
Self-regulation	Psilocybin	14.73	5.06	52
	Placebo	15.75	4.49	52
Noticing	Psilocybin	15.58	4.64	52
	Placebo	14.96	4.89	52
Emotional awareness	Psilocybin	19.42	6.84	52
	Placebo	20.67	5.22	52
Body listening	Psilocybin	9.12	4.14	52
	Placebo	9.77	4.31	52

MAIA: Multidimensional Assessment of Interoceptive Awareness Scale; SD: standard deviation.

#### Correlations of scales

We calculated the Pearson’s *r* correlations across subscales of the DASS-21 and MAIA per condition. Forty participants could be included in this analysis. The analysis was corrected for multiple comparisons using false discovery rate (see Tables 6 and 7).

**Table 6.** Psilocybin condition correlations between the different scale variables included in the study.

Variables	1	2	3	4	5	6	7
1. Depression	–	–	–	–	–	–	–
2. Anxiety	0.13	–	–	–	–	–	–
3. Stress	0.40*	0.47*	–	–	–	–	–
4. Body listening	0.19	0.22	0.40*	–	–	–	–
5. Emotional awareness	0.29	0.00	0.29	0.80*	–	–	–
6. Noticing	0.03	0.03	0.21	0.70*	0.75*	–	–
7. Self-regulation	0.25	0.24	0.42	0.62*	0.69*	0.56*	–

* Denotes significance level *p* < 0.05.

**Table 7.** Placebo condition correlations between the different scale variables included in the study.

Variables	1	2	3	4	5	6	7
1. Depression	–	–	–	–	–	–	–
2. Anxiety	0.23	–	–	–	–	–	–
3. Stress	0.39	0.65*	–	–	–	–	–
4. Body listening	−0.06	0.06	0.04	–	–	–	–
5. Emotional awareness	0.07	−0.13	0.07	0.46*	–	–	–
6. Noticing	0.15	−0.04	0.14	0.54*	0.67*	–	–
7. Self-regulation	−0.13	−0.04	−0.16	0.31	0.27	0.38	–

* Denotes significance level *p* < 0.05.

### Confirmatory analyses

#### DASS-21

**Figure 4.**
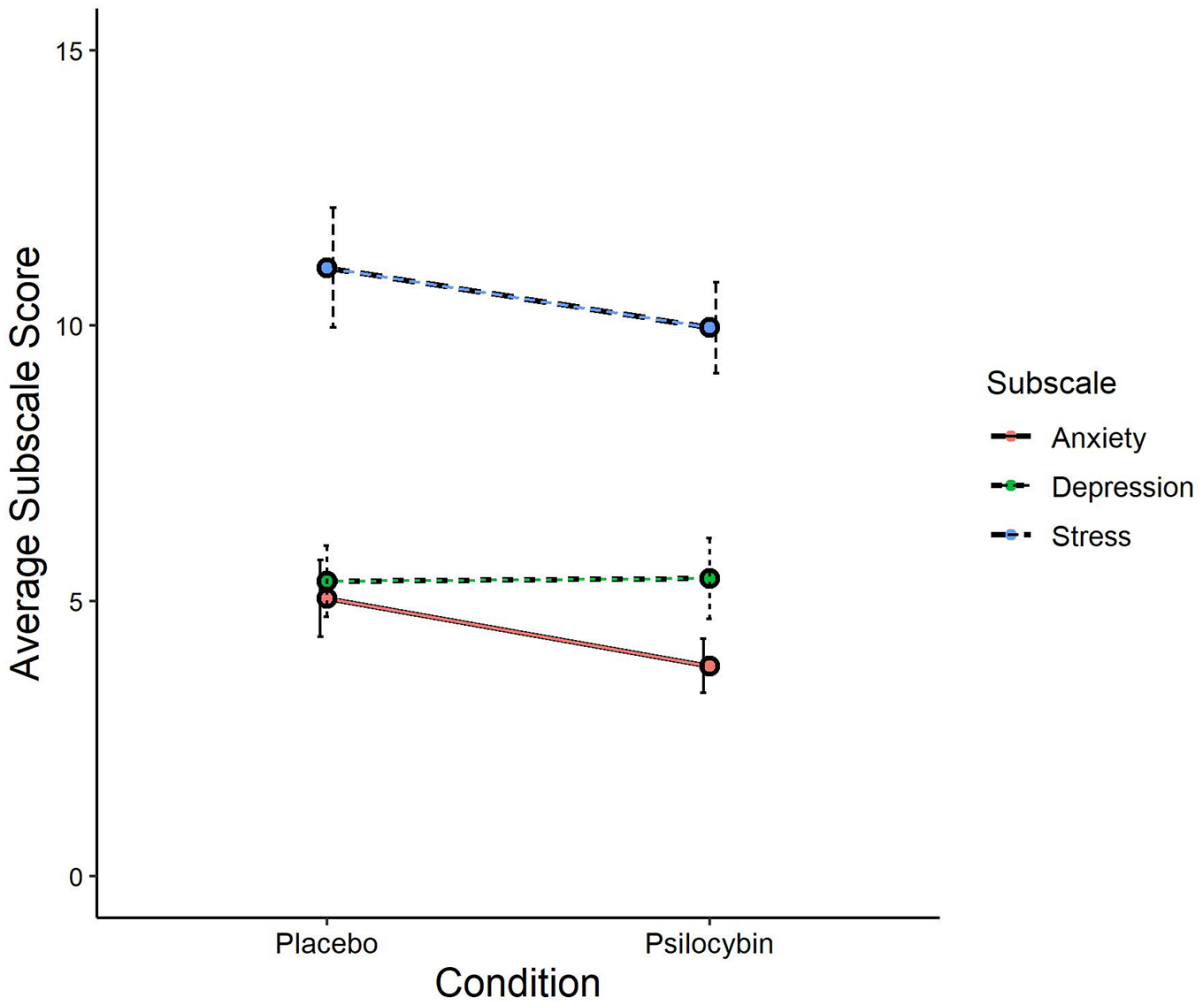
DASS-21 subscales score comparison between conditions. DASS-21: Depression Anxiety Stress Scale-21.

In contrast to our prediction for H1, the comparison between placebo and psilocybin conditions with depression and anxiety scores as the outcome variables did not reveal an effect of condition, *F*(1, 43) = 0.59, *p* = 0.45, η^2^ = 0.006. This was confirmed by the Bayesian statistic BF_10_ = 0.24, meaning the data were only 0.24 times more likely under the alternative hypothesis than under the null hypothesis and thereby suggesting moderate to strong evidence for the null hypothesis of no difference between placebo and psilocybin conditions in DASS-21 scores. Further subscale-specific analyses, which were Bonferroni corrected for two comparisons, did not find an effect of condition on depression scores, *t*(43) = 0.04, *p* = 1.00, *d* = 0.01, BF_10_ = 0.16, nor anxiety scores *t*(43) = 1.36, *p* = 0.36, *d* = 0.21, BF_10_ = 0.39. These condition comparisons are visually presented in Figure 4. The Stress subscale is presented in this figure as well, but the analysis is reported in the exploratory section.

Next, we added block-order to the rmANOVA to investigate whether there was a difference in scores between participants with respect to receiving the psilocybin dose in the first vs second block, but failed to find an interaction between block-order and condition, *F*(1, 42) = 0.64, *p* = 0.43, η^2^ = 0.01. Bayesian statistics support this result by revealing stronger evidence for the null hypothesis that block-order did not affect our experimental manipulation, BF_10_ = 0.034. There was also no main effect of block-order, *F*(1, 42) = 0.25, *p* = 0.62, η^2^ = 0.003, BF_10_ = 0.214.

#### Emotional go/no-go

The results revealed a main effect of emotion, *F*(5, 195) = 40.14, *p* < 0.001, η^2^ = 0.20, BF_10_ = 1.91e + 15, but no main effect of condition, *F*(1, 39) = 0.88, *p* = 0.35, η^2^ = 0.009, BF_10_ = 1.90. In contrast to our prediction for H2, the Emotion × Condition interaction analysis did not yield an effect, *F*(5, 195) = 0.37, *p* = 0.87, η^2^ = 0.002, which was confirmed by Bayesian statistics, BF_10_ = 0.15, suggesting stronger evidence for the null hypothesis that RTs did not differ between condition when emotional valences are taken into account. Including block-order as a between-subjects variable did not reveal an interaction with condition, *F*(1, 38) = 0.07, *p* = 0.79, η^2^ < 0.001, BF_10_ = 1.03, which indicates that there was no difference in RTs between participants who received psilocybin first and those who received the placebo first. The RTs are visually presented above in Figure 3.

#### H3

Due to lack of evidence for H1 and H2, we did not conduct a mediation analysis to investigate whether DASS-21 scores are mediated by emotional go/no-go RTs to negative emotional expressions.

### Exploratory analyses

#### Post hoc power analyses

We conducted a post hoc power analysis to investigate whether our emotional go/no-go task design was sufficiently powered to detect a condition with emotion interaction effect. This indicated that with our observed effect size, our study only achieved a power of 0.12. However, based on results by Kometer et al. (2012), who used a similar design to assess the effects of a full dose of psilocybin, we hypothesized that psilocybin would increase RTs for negative stimuli and we can further expect an increase in RTs to neutral stimuli. If we presume a 15% increase in our observed RTs to negative stimuli and a 10% increase in our observed RTs to neutral stimuli, this would translate to an effect size for the interaction effect between condition and emotion of *d* = 0.12. To detect this effect at *p* < 0.05 with a power of β = 0.96, 40 participants would be needed, which was the sample we used in this study. We implemented this analysis with ‘simulated’ RT data using the ANOVA-Power Shiny application (ANOVA_power, n.d.) and thus note that this power analysis remains speculative because we lack the data to obtain a sufficiently reliable effect size estimate that can be used as the input for such an analysis. Future studies could translate their hypotheses in expected RT patterns, to make better-informed decisions about the planned sample size.

#### MAIA

We used frequentist and Bayesian rmANOVA to compare psilocybin condition scores with placebo scores per subscale. We failed to find differences for any of the subscales assessed, including emotional awareness, *F*(1, 51) = 0.91, *p* = 0.35, η^2^ = 0.02, BF_10_ = 0.37; for body listening, *F*(1, 51) = 0.53, *p* = 0.47, η^2^ = 0.01, BF_10_ = 0.27; self-regulation, *F*(1, 51) = 1.05, *p* = 0.31, η^2^ = 0.02, BF_10_ = 0.38 and noticing, *F*(1, 51) = 0.39, *p* = 0.54, η^2^ = 0.01, BF_10_ = 0.26. However, there was a significant main effect of block-order, but not in interaction with condition, for nearly all subscales, including emotional awareness, *F*(1, 50) = 21.49, *p* < 0.001, η^2^ = 0.12, BF_10_ = 24.31; body listening, *F*(1, 50) = 12.74, *p* < 0.001, η^2^ = 0.08, BF_10_ = 5.57 and self-regulation, *F*(1, 50) = 9.96, *p* = 0.003, η^2^ = 0.07, BF_10_ = 3.51. This main effect of block-order for noticing was found in frequentist but not Bayesian statistics, *F*(1, 50) = 9.91, *p* = 0.003, η^2^ = 0.07, BF_10_ = 3.81.

**Figure 5.**
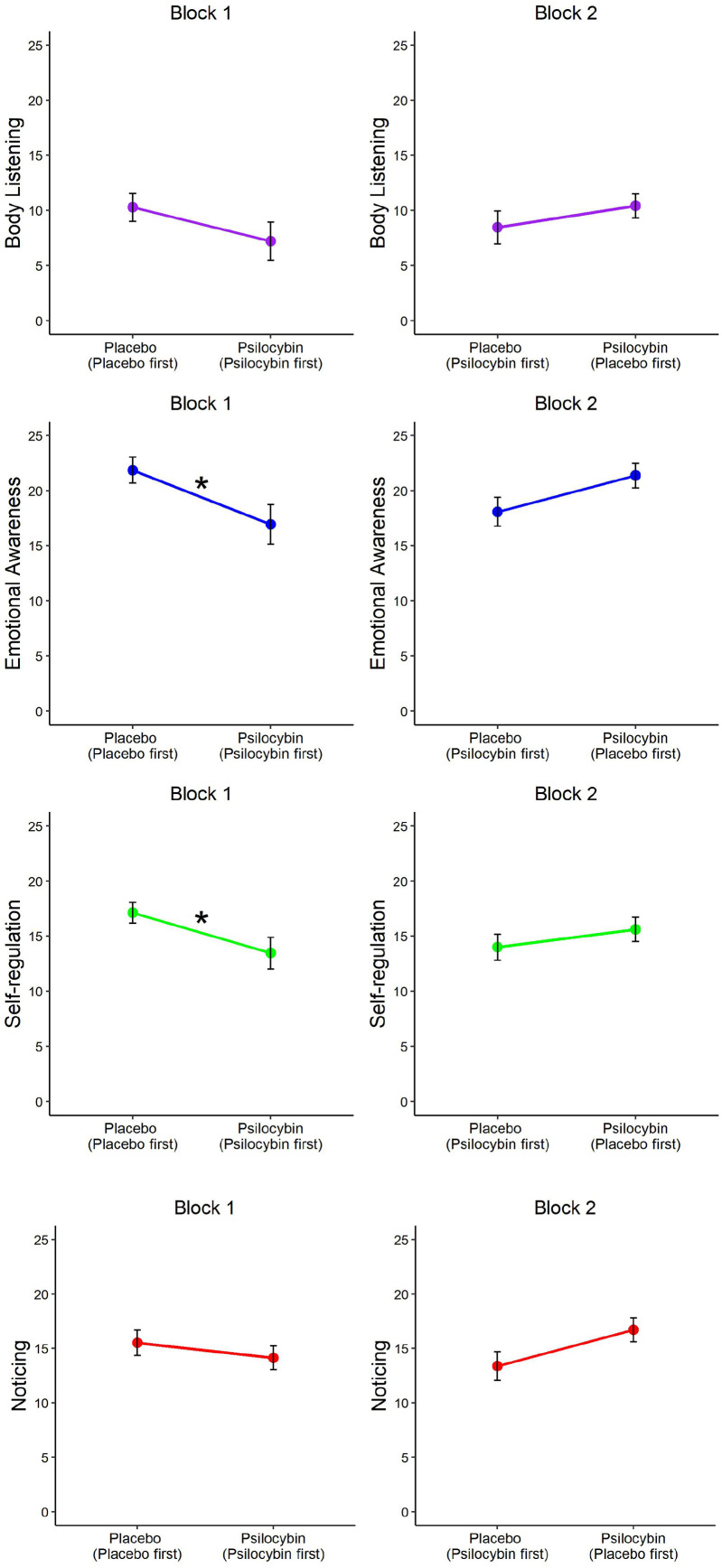
MAIA subscale score comparison between conditions, per block and block-order group. MAIA: Multidimensional Assessment of Interoceptive Awareness Scale. The line colours are used to visually differentiate between subscales. *Denotes significance level *p* < 0.05.

Further independent-samples *t* tests per block, Bonferroni corrected for 8 comparisons, revealed significant differences between conditions in block 1 for emotional awareness, *t*(62) = 3.08, *p* = 0.024, *d* = 0.78, BF_10_ = 12.02, and self-regulation, *t*(62) = 3.19, *p* = 0.02, *d* = 0.80, BF_10_ = 15.85, but not for the body listening and noticing subscales. Analyses for block 2 did not reveal significant differences between conditions. Figure 5 reveals that those in the placebo condition in block 1, meaning the placebo-first block-order group, scored higher on emotional awareness and self-regulation relative to the psilocybin condition. A similar trend can be visually detected in block 2, but no longer reaches significance. We conducted paired-sample *t* tests to identify whether the subscale scores of the block-order groups changed significantly from block 1 to block 2. We found that the average subscale scores of the psilocybin-first group increased, and the placebo-first group decreased from block 1 to block 2, but the change in scores did not reach significance for either subscale.

#### DASS-21

Since we obtained baseline scores for each DASS-21 subscale and we obtained stress subscale scores for each measurement session, we explored these data in the following analyses, including 40 participants that attended all sessions and adequately followed the behavioural guidelines. The comparison between baseline, placebo and psilocybin conditions regarding depression, anxiety and stress subscale scores revealed a significant difference between conditions, *F*(2, 78) = 14.03, *p* < 0.001, η^2^ = 0.109, BF_10_ = 1.44e + 6, as well as a significant difference between subscales, *F*(2, 78) = 58.02, *p* < 0.001, η^2^ = 0.24, BF_10_ = 1.39e + 17, but no effects of block-order. We already knew from our confirmatory analyses that this difference between conditions is likely not driven by the comparison between placebo with psilocybin conditions for the anxiety and depression subscales. We thus explored whether this also pertains to the stress subscale by conducting a paired-samples *t* test assessing the difference between the psilocybin and placebo condition, using the same set of participants as in our confirmatory analyses for depression and anxiety (*n* = 44). We failed to find a difference between psilocybin and placebo conditions in the stress subscale, *t*(43) = 0.77, *p* = 0.45, *d* = 0.12, BF_10_ = 0.22 (see Figure 4).

This suggests that the effect of condition may be driven by baseline scores. We conducted paired-samples *t* tests to compare the psilocybin condition scores and the placebo condition scores separately with the baseline condition, per subscale and Bonferroni-adjusted for 6 comparisons. We found significant differences for almost all comparisons. In the comparison between baseline and the psilocybin condition, the difference for depression was not significant, *t*(39) = 2.62, *p* = 0.07, *d* = 0.42, BF_10_ = 3.40, but it was significant for anxiety, *t*(39) = 3.60, *p* < 0.001, *d* = 0.57, BF_10_ = 34.36, and for stress, *t*(39) = 4.76, *p* < 0.001, *d* = 0.75, BF_10_ = 803.22. In the comparison between baseline and the placebo conditions, the difference for depression was significant, *t*(39) = 3.97, *p* < 0.001, *d* = 0.63, BF_10_ = 89.49, as for stress, *t*(39) = 4.03, *p* < 0.001, *d* = 0.64, BF_10_ = 106.25, but not for anxiety, *t*(39) = 2.737, *p* = 0.054, *d* = 0.43, BF_10_ = 4.34.

These results suggest that scores differ by measurement time-point. To test this, we conducted a 3 × 3 rmANOVA with measurement time-points (baseline, block 1 and block 2) and subscales (depression, anxiety, stress) as within-subject factor. We found a significant difference between measurement time-points, *F*(2, 80) = 14.44, *p* < 0.001, η^2^ = 0.109, BF_10_ = 4.60e + 6.

**Figure 6.**
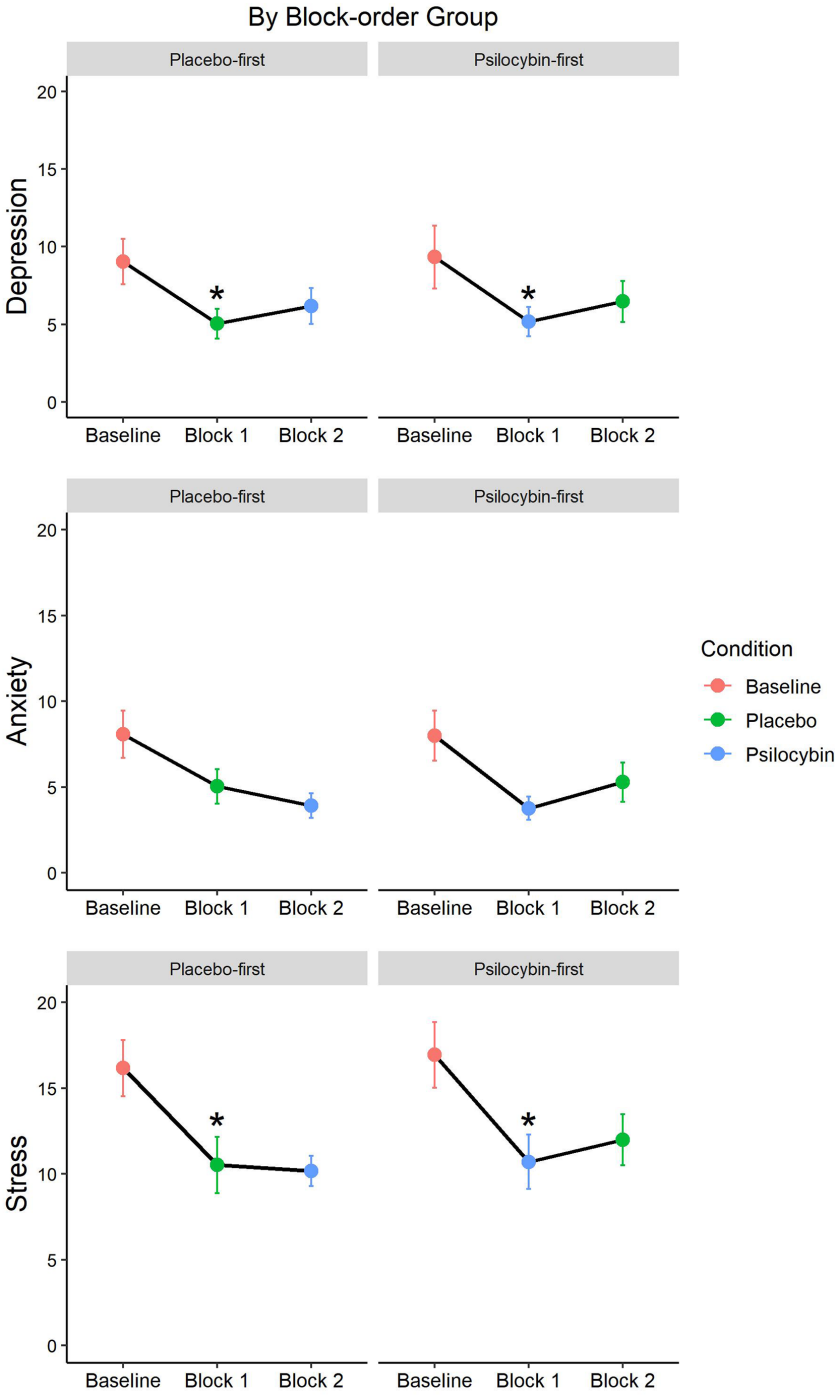
DASS-21 subscale condition comparisons with baseline per block and block-order group. DASS-21: Depression Anxiety Stress Scale-21. *Denotes significance level p < 0.05.

Paired-samples *t* tests, Bonferroni-adjusted for 12 comparisons, revealed a significant difference between baseline and block 1 for the depression and stress subscales, regardless of condition, but not for the anxiety subscale. The analysis did not reveal any significant differences between baseline and block 2 subscale scores. See Table 8 for the details of this analysis and see Figure 6 for a visual presentation of the depression, anxiety and stress subscale scores, respectively.

**Table 8.** DASS-21 paired-sample *t* tests comparing baseline to condition per block, block-order and subscale.

	Block-order	Subscale	Measure 1	Measure 2	*t*	*df*	*p*	Cohen’s *d*	BF_10_
Block 1	Psilocybin first	Depression	Baseline	Psilocybin condition	3.42	19	0.04	0.76	14.62
	Placebo first	Depression	Baseline	Placebo condition	3.71	28	<0.001	0.69	36.57
	Psilocybin first	Anxiety	Baseline	Psilocybin condition	3.04	19	0.08	0.681	7.19
	Placebo first	Anxiety	Baseline	Placebo condition	3.04	28	0.06	0.57	8.12
	Psilocybin first	Stress	Baseline	Psilocybin condition	4.57	19	<0.001	1.02	142.99
	Placebo first	Stress	Baseline	Placebo condition	4.07	28	<0.001	0.76	84.43
Block 2	Psilocybin first	Depression	Baseline	Placebo condition	2.45	19	0.29	0.55	2.5
	Placebo first	Depression	Baseline	Psilocybin condition	1.32	28	1.00	0.25	0.43
	Psilocybin first	Anxiety	Baseline	Placebo condition	2.01	19	0.62	0.46	1.36
	Placebo first	Anxiety	Baseline	Psilocybin condition	2.99	28	0.07	0.55	7.22
	Psilocybin first	Stress	Baseline	Placebo condition	2.78	19	0.14	0.62	4.45
	Placebo first	Stress	Baseline	Psilocybin condition	3.0	28	0.07	0.56	7.4

DASS-21: Depression Anxiety Stress Scale-21.

#### Sex differences

We explored whether the above results differ according to sex. We failed to find an interaction of sex with condition for the DASS-21, *F*(1, 42) = 0.10, *p* = 0.75, η^2^ = 8.26e − 4, BF_10_ = 0.09, nor for any of the MAIA subscales. In the emotional go/no-go task, we also failed to find an interaction of sex with condition and emotion, *F*(6, 228) = 0.58, *p* = 0.75, η^2^ = 0.002, BF_10_ = 0.026, nor any other effect of sex.

#### Condition identifications

The chi^2^ analysis of the guessed condition with actual condition contingency table revealed that participants could not accurately identify their condition above chance following block 1, χ^2^(2) = 4.89, *p* = 0.09. However, they did identify their condition following block 2, χ^2^(2) = 13.71, *p* = 0.001, meaning that participants broke blind during the second block of this study (see Table 9). These results are supported by Bayesian statistics which revealed more evidence for participants not identifying their condition in block 1, BF_10_ = 1.67, and more evidence for participants being able to identify their condition in block 2, BF_10_ = 268.84. We asked participants to guess their conditions after completing each block; thus, it is not clear whether they broke blind before S3 or S4.

**Table 9.** Condition contingency table.

Guess block 1	Condition		Total
	Psilocybin	Placebo	
Yes	9 (19.57%)	4 (8.7%)	13 (28.26%)
Maybe	6 (13.04%)	7 (15.22%)	13 (28.26%)
No	6 (13.04%)	14 (30.44%)	20 (43.48%)
Total	21 (45.65%)	25 (54.35%)	46 (100%)
Guess block 2	Psilocybin	Placebo	Total
Yes	12 (40%)	2 (6.67%)	14 (46.67%)
Maybe	1 (3.33%)	2 (6.67%)	3 (10%)
No	2 (6.66%)	11 (36.66%)	13 (43.33%)
Total	15 (50%)	15 (50%)	30 (100%)

#### Expectation effects

To explore whether participants’ beliefs regarding their condition assignment influenced their responses to the above measures, we analysed the between-subject effect of expected condition per block and per subscale score of the DASS-21 and MAIA. We found a significant effect of guessed condition for the MAIA subscale ‘emotional awareness’ in block 1, *F*(2, 41) = 4.11, *p* = 0.02, η^2^ = 0.17. However, this was not strongly supported by Bayesian statistics, BF_10_ = 2.95, nor did it survive Bonferroni correction for the 8 comparisons of the MAIA subscales per block (*p* = 0.19).

Next to expected condition, we also assessed perceived drug strength by asking participants how strongly they felt the drug effects on a scale from 0 to 100. We also conducted a linear regression to assess the effect of perceived drug strength per subscale score per block. Here again we found only one effect for the MAIA subscale Body Listening in Block 2, *F*(2, 53) = 5.93, *p* = 0.02, but this was also not supported by Bayesian statistics, BF_10_ = 1.33, nor did it survive Bonferroni correction (*p* = 0.14). Thus, no significant differences between expected drug conditions nor effects of perceived drug strength were found for any of the DASS-21 nor MAIA subscales.

#### Reported subjective effects

We asked those who expected that they were in the psilocybin microdose condition to write freely which subjective effects they experienced that led them to guess this condition assignment. We list these reports per actual condition assignment and per block, both in their original form (see Table 10 for block 1 and Table 11 for block 2) and categorized with the frequency of reported effects across participants (see Table 12 for block 1 and Table 13 for block 2).

**Table 10.** Block 1 reported subjective effects of expected psilocybin condition, per assigned condition.

Psilocybin condition
‘Higher energy, emotional highs’.
‘Slightly more clear-minded/concentrated/happy . . . thought I noticed things that I’m familiar with from earlier truffle trips after the first dose’.
‘On some occasions, altered vision, slightly nauseated feeling, buzz feeling in the body, generally quite positive mood, thinking felt somewhat divergent’.
‘Physical tension, nervousness, accelerated thinking, general feeling of psychedelic come up’.
‘A “lightness to it all”, electrical sensations in my jaw, a slight “rollercoaster” feeling in tummy sometimes, a wave of tiredness about 4–6 hours later’.
‘I felt high a bit, weird dreams, good sleep, more calm at work’.
‘Happier, more alert and aware, far more sensitive to other stimulants’.
‘Suddenly extremely positive and hopeful after a severely depressive 3 months; different sleep patterns; jittery different feeling for some hours after taking the pills’.
‘Dizzy, increased emotions, super dry mouth, increased focus’.
Placebo condition
‘Giddiness, pattern making’.
‘I felt a bit aroused and giggly, excited’.
‘Increased focus, interesting thoughts, more self-reflection’.
‘Micro effects of what a normal psilocybin trip is. Sense of self, sense of colour, felt more in touch with surrounding’.

**Table 11.** Block 2 reported subjective effects of expected psilocybin condition, per assigned condition.

Psilocybin condition
‘Things around me seemed different’.
‘I was very giggly, laughing about everything more than usual. Also I was overall quite happy’.
‘Tiredness, feeling a little high. Heightened urge to be in nature. More creativity and better concentration. More self-reflection’.
‘First day I felt a lot of euphoria, feeling of oneness and flow. Days after I had to work, so couldn’t really relax into it but felt I could breathe more deeply’.
‘Heightened focus, more energy/ less sleepy, more positive’.
‘I felt like I became a bit trip during both tests at the lab. With the 3d dose I took at home I had to stop working (I work from home) and lay on my bed. I was seeing shapes and felt energy in or around my womb. After the last dose I felt extremely peaceful and in love with life. Walking on the street I felt like I wanted to tell so many people how beautiful they looked. And I was sighing and breathing very deep, this usually only happens like this when I take shrooms or MDMA’.
‘A slight euphoric effect after taking a dose. Definitely an urge to feel more relaxed and explorative’.
‘I felt slightly more energized during the second part of the trial’.
‘I felt like some energy was flowing through my body making me want just to lay down and relax’.
‘I could feel a warm sensation in my stomach. I felt a bit different, also more lazy. I could really feel it coming up’.
‘Increased happiness, More empathy, More self-reflection, Energetic high in body, Sweaty hands, More easily distracted by external events’.
‘More attentive to surrounding details, Increased tolerance and patience, less irritation during the day, slightly elevated mood’.
Placebo condition
‘Little bit high, different concentration’.
‘Pressure behind eyes. Light-headed’.

**Table 12.** Block 1 reported subjective effects categorized with reported frequency across participants, per condition.

	Psilocybin	Placebo
Affective		
Happier	4	
Emotional highs	2	
Giddy		2
Calm	1	
Nervous	1	
Cognitive		
More concentration/focus	2	1
Clear minded	1	
Accelerated thinking	1	
Divergent thinking	1	
Pattern making		1
Alert	1	
Self-reflective		1
Interesting thoughts		1
Physical/sensory		
Enhanced sensory perception	1	1
Dizziness	1	
Nausea	1	
Dry mouth	1	
More energy	1	
Jittery	1	
Aroused		1
Wave of tiredness 4–6 h after ingestion	1	
Electrical sensations in jaw	1	
Buzz feeling in body	1	
Rollercoaster feeling in stomach	1	
Tension	1	
Better sleep	1	
Change in sleep patterns	1	
Other		
Effects that liken previous psychedelic experience	2	1
Weird dreams	1	
Feeling ‘high’	1	
More sensitive to other stimulants	1	
More in touch with surroundings		1

**Table 13.** Block 2 reported subjective effects categorized with reported frequency across participants, per condition.

	Psilocybin	Placebo
Affective		
Happier /more positive	4	
Euphoria	2	
Peaceful	1	
Flow	1	
Feeling love	1	
Giggly	1	
Less irritated	1	
Lazy	1	
Empathetic	1	
Patient/tolerant	1	
Cognitive		
Self-reflective	2	
More concentration/focus	2	
Creative	1	
Distracted	1	
Explorative	1	
Attention to detail	1	
Physical/sensory		
Feeling energy/sensations in in the body	4	
Enhanced/altered sensory perception	2	
Feeling more energized	2	
Deeper breathing	2	
Relaxed	1	
Pressure behind eyes		1
Tired	1	
Sweaty hands	1	
Light-headed		1
Other		
Feeling ‘high’/feel it ‘coming up’	2	1
Effects that liken previous psychedelic experience	1	
Urge to be in nature	1	

## Discussion

We conducted this study to investigate the effect of repeated psilocybin microdosing on depression and anxiety symptoms, emotion processing and interoceptive awareness. We hypothesized that psilocybin microdosing would reduce symptoms of anxiety and depression, increase the processing time needed to identify negative emotions and increase interoceptive awareness. Our results suggest that the psilocybin microdose did not affect interoceptive awareness, but that there was an effect of the block-order variable. In block 1, the psilocybin-first block-order group had a lower average on two subscales scores of the MAIA compared with the Placebo-first block-order group, but this difference was no longer significant in block 2. We explored the possibility that the psilocybin-first block-order group’s interoceptive awareness increased due to repeated psilocybin microdosing after block 1 and therefore achieved higher scores in block 2 which were more similar to the placebo-first group. However, although there was an increase in average score, this change was not significant. It could well be that the block-order groups already differed in interoceptive awareness at baseline and became more similar in their scores over time regardless of condition assignment – however as we did not assess baseline scores on interoceptive awareness, this possibility cannot be verified.

The effect of repeated microdosing on emotion processing, as measured using an emotion go/no-go task, and symptoms of anxiety, depression and stress also did not differ from placebo. Our finding that psilocybin microdosing does not affect symptoms of anxiety and depression contradicts previous survey studies which reported marked reductions in negative emotionality following the repeated microdosing of psychedelic substances (Anderson et al., 2019; Johnstad, 2018; Polito and Stevenson, 2019). This discrepancy may be due to four key elements in our method, including the mental well-being of our participants at baseline, the use of psilocybin only, the duration of the microdosing period and the inclusion of a placebo condition.

First, our participants were only admitted if our pre-trial screening deemed them as physically and mentally healthy, and their symptoms of anxiety and depression at baseline were within the normal range on average. Johnstad (2018) and Anderson et al. (2019) did not include this criterion within their designs, allowing for participants with clinical range symptoms at baseline. This creates the possibility that their significant reductions in negative emotionality were in part due to higher negative emotionality at baseline, whereas our participants may have experienced a ceiling effect; they were already mentally healthy prior to microdosing and could not show further improvement during the study. However, this argument is countered by Polito and Stevenson (2019) who did explicitly focus on a non-clinical population. They reported low DASS-21 scores at baseline, yet found marked reductions in depression and stress scores. Thus, although the baseline DASS-21 scores of our participants were in the same range as those of Polito and Stevenson (2019), we failed to see additional improvements in our participants’ depression scores after microdosing. However, Polito and Stevenson (2019) did not find a reduction in anxiety scores after microdosing, which does align with our findings. Moreover, post hoc analyses of the DASS-21 subscale scores in comparison with baseline scores did reveal significant reductions in the stress and depression subscales in block 1, but regardless of condition.

Second, previous results demonstrated by Johnstad (2018), Anderson et al. (2019) and Polito and Stevenson (2019) were based on psychedelic microdoses in general, while we chose to focus on specifically psilocybin. It is thus possible that previous results were driven by the effects of psychedelic substances other than psilocybin. However, Bershad and colleagues (2019) investigated the effect of three different microdoses of LSD (6.5, 13 and 25 μg) and also did not find a significant effect of these doses on emotion processing nor on negative emotionality. Relatedly, in our study, we had little control over the specific amount of psilocybin that participants consumed, due to natural variability in different batches of psilocybin-containing truffles. Next to that, it is possible that we also manipulated other active compounds found in the psilocybin-containing truffles and that these influenced our results.

Third, our participants consumed the microdoses for a shorter duration (3 weeks) compared with those in the research by Polito and Stevenson (2019; 6 weeks) and likely in the research by Johnstad (2018) and Anderson et al. (2019), although here the specific duration of microdosing was not reported. While the appropriate time necessary for the benefits of microdosing to take effect is unknown, it is known that serotonergic antidepressants can take up to 2 months before measurable effects arise (Harmer et al., 2009), potentially because their effects are due to certain downstream changes in brain structure and function (Erb et al., 2016; Hanson et al., 2011). The argument that effects of microdoses may also require a longer period of repeated dosing rests on two key findings: that depression and stress-related disorders are associated with neural atrophy in the prefrontal cortex (PFC; Christoffel et al., 2011) and that serotonergic psychedelics can increase structural and functional plasticity in the PFC (Ly et al., 2018; Olson, 2018), thereby potentially counteracting the neurobiological markers of these disorders. It is possible that a period of consistent microdosing which succeeds 3 weeks is required for such changes to develop and we can expect an effect on emotion processing and mood-related symptoms only after these changes have occurred.

Nevertheless, Cameron et al. (2019) administered microdoses of the serotonergic psychedelic DMT to rats every third day for 7 weeks and revealed no markers of increased neural plasticity. In fact, the researchers found a decrease in dendritic spine density in PFC of female rats. Important to note is that the original association between serotonergic psychedelics and neuronal plasticity is based on the effect of a single large serotonergic psychedelic dose. Single large doses of DMT and LSD were found to promote spinogenesis, synaptogenesis and neural plasticity in cortical neuron cultures of rats 24 h after administration (Ly et al., 2018). Taken together, this evidence, although limited in its generalizability to humans, may indicate that regardless of the duration of the dosing period, psilocybin microdoses are simply not potent enough to trigger structural changes in the cortex.

Another factor that could account for the apparent conflict with previous findings is our implementation of a placebo-control group. It is plausible that previous results were driven by participants’ expectations rather than the chemical components of the doses. This would mean that the sole act of taking doses improved participants’ mental health scores, regardless of whether the doses were placebos or psilocybin microdoses. Indeed, Kaertner et al. (2021) found that positive expectancy scores at baseline predicted changes in well-being after 4 weeks of microdosing. In our participants, we observed an overall decrease in the depression and stress scores from baseline to block 1, irrespective of the condition that the participants were assigned to. This effect, termed the ‘placebo effect’, is especially relevant in clinical and pharmacological research and refers to the situation when blinded participants in the placebo condition experience a reduction in symptoms either due to their positive expectations towards the treatment condition or due to previous conditioning of the treatment condition (Meissner et al., 2011). Especially in antidepressant research, placebo doses evoke reductions in symptoms comparable with the antidepressant (Kirsch, 2014). Such potent placebo responses may also be contributing to previous findings regarding the effects of psilocybin microdosing and make it difficult to assess whether psilocybin microdosing is effective beyond expectations and conditioning.

Of relevance, through an exploratory analysis of our participants’ condition guesses, we found that participants broke blind regarding their condition in the second block. This confound had the potential to further contribute to response expectancy effects. However, we found no difference between psilocybin and placebo conditions in our outcome measures in either block, which indicates that explicit expectations likely did not influence our results. Moreover, in contrast to Kaertner et al. (2021), we found no effect of expectation in further post hoc exploratory analyses. We propose two possible reasons for this lack of an effect of psilocybin microdosing on outcome measures: either the placebos and psilocybin microdoses were equally ineffective at influencing a change in response to the scales that we used (potentially the measures were not sensitive enough), or the placebo effect was equally as effective as the microdosing effect but was guided by processes other than explicit expectations, such as previous conditioning (e.g. as demonstrated by Amanzio and Benedetti, 1999). Previous experience with psychedelic substances could evoke a placebo effect based on conditioning. Most participants in this study had taken psychedelics before and may therefore have been subject to such conditioning. Thus, including a placebo condition may have dampened our effect of interest.

Finally, we need to consider the possibility that microdosing does not affect depression and anxiety at all, as our findings consistently indicate. Previously reported beneficial effects may be related to other confounding factors, as mentioned above, and experimental research thus far fails to show these hypothesized effects of microdosing on clinically relevant outcome measures (Bershad et al., 2019; Family et al., 2020).

## Limitations

We note five key limitations of our study. First, our sample suffers from selection bias, since participants were self-selected from a microdosing workshop. As a result, most of our participants had tried psychedelics previously, which means that they may have broken blind easier or may have been desensitized to the microdosing effects. Second, the psilocybin doses were made by the participants using dried psilocybin truffles, meaning that we cannot be sure of the exact amounts of psilocybin in the individual doses that the participants consumed. It is possible that the degree of psilocybin content varied across participants and thereby obscured our results. Third, we encountered a large drop-out rate during this project and several participants did not sufficiently comply with the behavioural guidelines to be included in the analyses. This resulted in small sample size relative to existent observational studies and in a further selection bias (i.e. only motivated participants likely stayed in). Moreover, due to such sample size, our study may have been underpowered to detect true effects, particularly the interaction effect hypothesized for the emotional go/no-go task. Our post hoc power analysis suggested that our design, given our observed data, was insufficiently powered to detect this effect. Simulated data in the hypothesized direction, however, yielded sufficient power with a large effect size. Of course, as noted earlier, this analysis based on simulated data remains speculative and we encourage future studies to plan their sample size according to expected RT patterns. Fourth, we measured the effects in our study only after self-administration of a dose, and not between doses or after each block. Thus, our results may be confounded by the acute effect of the psilocybin dose, which may differ from its persistent effect after the acute chemical-induced symptoms have subsided. However, Szigeti et al. (2021) did assess both acute and post-acute effects and found no significant microdose vs placebo differences in psychological outcomes when accounting for participants breaking blind. Last, our study is a combined field and lab-based study, meaning that the results may not be readily generalizable or replicable, for example, in a more clinical setting.

## Conclusion and suggestions for future research

Our study did not find an effect of psilocybin microdosing on interoceptive awareness, nor on emotion processing or symptoms of anxiety and depression. Furthermore, we found that participants easily break blind regarding their experimental condition in a within-subjects design, despite the use of small dosages. This confound needs to be considered in future studies to more reliably establish the potential promises and pitfalls of using psychedelic microdosing.
